# Research progress on the immune microenvironment and immunotherapy in gastric cancer

**DOI:** 10.3389/fimmu.2023.1291117

**Published:** 2023-11-23

**Authors:** Pei Mou, Qing-hua Ge, Rong Sheng, Teng-fei Zhu, Ye Liu, Kai Ding

**Affiliations:** ^1^ Changzheng Hospital of Naval Medical University, Shanghai, China; ^2^ Department of Otolaryngology, Changzheng Hospital of Naval Medical University, Shanghai, China; ^3^ Department of Outpatient, Changzheng Hospital of Naval Medical University, Shanghai, China; ^4^ Department of Anesthesiology, Changzheng Hospital of Naval Medical University, Shanghai, China; ^5^ Department of Blood Transfusion, Changzheng Hospital of Naval Medical University, Shanghai, China; ^6^ Department of Gastroenterology, Changzheng Hospital of Naval Medical University, Shanghai, China

**Keywords:** gastric cancer, immune microenvironment, immunotherapy, immunity, single-cell sequencing

## Abstract

The tumor microenvironment, particularly the immune microenvironment, plays an indispensable role in the malignant progression and metastasis of gastric cancer (GC). As our understanding of the GC microenvironment continues to evolve, we are gaining deeper insights into the biological mechanisms at the single-cell level. This, in turn, has offered fresh perspectives on GC therapy. Encouragingly, there are various monotherapy and combination therapies in use, such as immune checkpoint inhibitors, adoptive cell transfer therapy, chimeric antigen receptor T cell therapy, antibody-drug conjugates, and cancer vaccines. In this paper, we review the current research progress regarding the GC microenvironment and summarize promising immunotherapy research and targeted therapies.

## 1 Introduction

Gastric cancer (GC) is one of the most pervasive malignancies of the digestive system, with the fifth highest incidence rate and fourth highest mortality worldwide (1). However, for inoperable advanced-stage patients, mortality rates remain concerning. In addition, no effective treatments are currently available (2). Irrespective of the histopathological or molecular subtype, GC does not exist as an independent cluster of cancer epithelial cells. Instead, these tumor cells possess an intricate morphology with various resident cells around the tumor microenvironment (TME). This TME contains multiple cellular categories, such as immune cells, endothelial cells, and fibroblasts (3, 4). An increasing number of researchers have realized that the cellular characteristics of the TME, especially those of the immune cells, play a prominent part in tumor proliferation and metastasis. Thus, there is an urgent need to intensify the comprehension of the TME, especially the tumor immune microenvironment (TIME), to identify new targets for enhancing the clinical efficacy of GC therapy. In this review, we present a comprehensive summary of the GC immune microenvironment, as well as the current landscape of immunotherapy and targeted therapy for GC.

## 2 Immune microenvironment of gastric cancer

**Figure 1:**
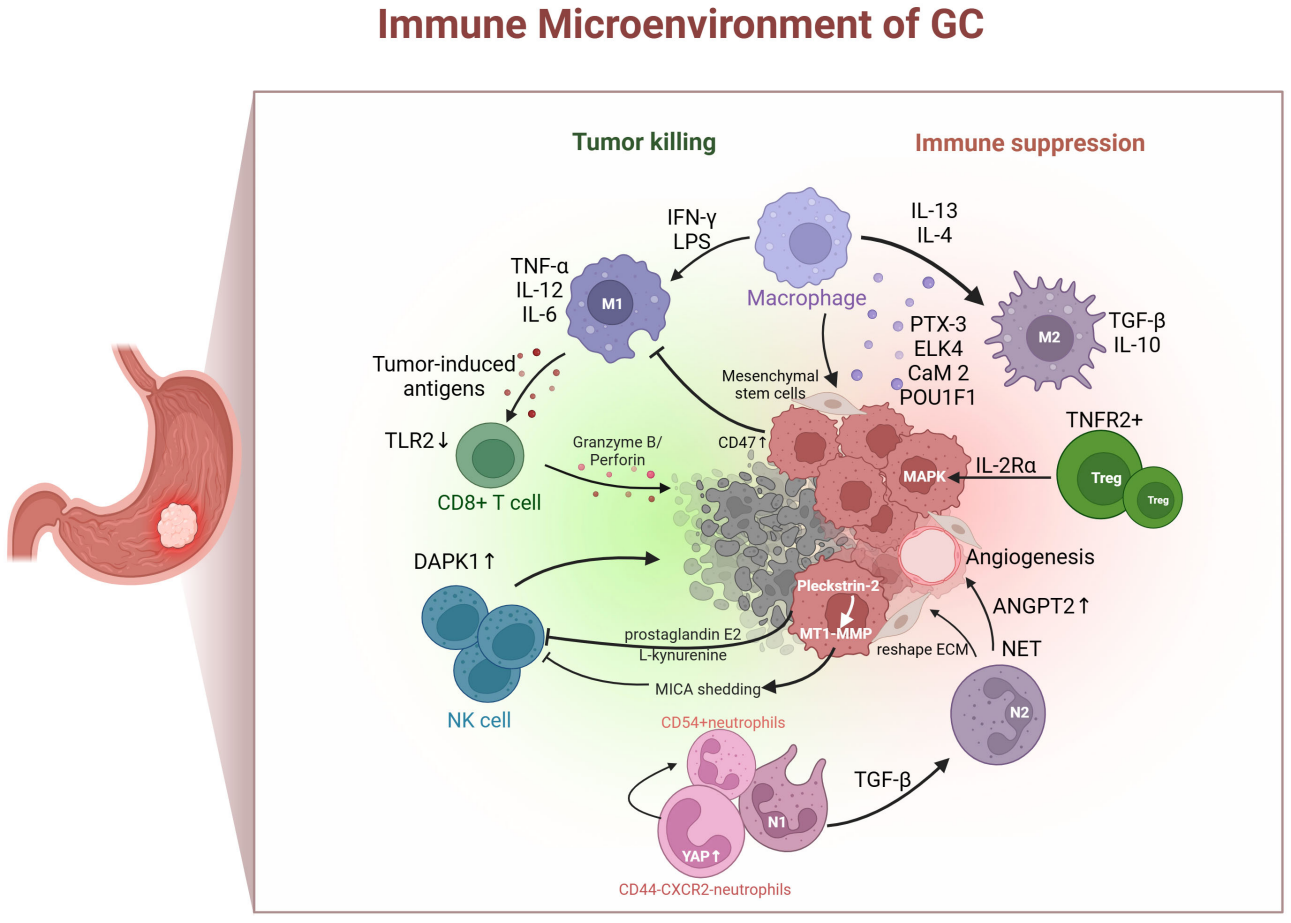
Immune cells (tumor-associated neutrophils, macrophages, T cells, and NK cells) in gastric cancer immune microenvironment. IFN-γ, interferon-gamma; LPS, lipopolysaccharide; IL, interleukin; TGF-β, transforming growth factor β; PTX-3, pentraxin-3; ELK4, ETS-like transcription factor 4; CaM 2, calmodulin 2; POU1F1, POU class 1 homeobox 1; Treg, regulatory T cell; TNFR2, tumor necrosis factor receptor 2; MAPK, mitogen-activated protein kinase; ANGPT2, angiopoietin-2; NET, neutrophil extracellular traps; ECM, extracellular matrix; MT1-MMP, membrane-type 1 matrix metalloproteinase; MICA, major histocompatibility complex class I chain-related A; YAP, yes-associated protein; DAPK1, death-associated protein kinase 1;TLR2, toll-like receptor 2; TNF-α, tumor necrosis factor-alpha.

The TIME plays a crucial role in tumor progression, invasion, metastasis, immune evasion, and therapy resistance (5, 6). The stomach possesses potent acidic conditions and a distinctive endocrine system, which makes the TIME of GC disparate (7, 8). Tumors use diverse mechanisms to evade immune surveillance. These include enhancing negative immune regulatory mechanisms and impacting antigen presentation. Immune cell populations, such as tumor-associated macrophages lymphocytes, tumor-associated neutrophils, T cells, natural killer (NK) cells, play pivotal roles in gastric tumorigenesis through regulating the immune responses. Consequently, the anti-tumor capacity of immune cells in the GC is impaired, affecting the likelihood of mounting an effective anti-tumor response (**Figure 1**).

### 2.1 Tumor-associated macrophages

Traditionally, macrophages infiltrating the TME were thought to exist in two polarized activation states. One is the classical M1-like macrophage, induced by interferon-gamma and lipopolysaccharide, which expresses tumor necrosis factor-alpha, interleukin-12, and interleukin-6, and has a tumor-inhibitory function. The other is the M2-like macrophage, induced by interleukin-13 and interleukin-4, expressing transforming growth factor β (TGF-β) and interleukin-10, which plays a role in promoting tumor growth (9, 10). Several components of the GC TME participate in M2 polarization, including pentraxin-3, ETS-like transcription factor 4, calmodulin 2, and POU class 1 homeobox 1 (11–14). Furthermore, M1-like macrophages exhibit a potent phagocytic capacity by decreasing tumor cell numbers and delivering tumor-induced antigens to T cells (15). However, tumor cells can evade phagocytosis by increasing the expression of anti-phagocytic markers such as CD47 (16). Mesenchymal stem cells are induced by macrophages, with the aim of acquiring tumor-related fibroblast-like characteristics and proinflammatory phenotypes, thereby changing the inflammatory microenvironment and promoting the cancerous transformation of gastric epithelial cells (17). Currently, macrophages are an emerging target for tumor therapy, which includes inhibiting the recruitment of macrophages in tumors, depleting their numbers, inducing macrophages to reprogram to the M1 phenotype, and enhancing phagocytosis (18). As a new model, chimeric antigen receptor (CAR) macrophages have been used in stage I clinical experiments, but their application in GC treatment is relatively limited (19).

### 2.2 Tumor-associated neutrophils

Tumor-associated neutrophils (TANs) are functionally divided into N1 cells that suppress tumors and N2 cells that promote tumors (20, 21). A retrospective study showed that vast quantities of TANs infiltrate GC tissue, suggesting a greater risk of lymph node metastasis (22). TGF-β in the TME can induce N1 to N2 polarization (23). Moreover, TANs facilitate immune tolerance by reshaping the extracellular matrix, boosting angiogenesis, producing neutrophil extracellular traps, and interacting with other immune cells (24). Neutrophil extracellular traps deployed by neutrophils may induced angiopoietin-2 overexpression in GC TME that promote the remodeling of vascular and facilitate tumor progression (25, 26). A Hippo regulon with unique YAP signature genes was detected in CD44^–^CXCR2^–^ neutrophils, and activating YAP could promote the differentiation into CD54^+^ neutrophils and enhance their antitumor activity (27). Although a few treatment options target neutrophils to ease immune tolerance, decreasing the infection risk resulting from neutrophil levels has become the largest barrier to this regimen.

### 2.3 T cells

T cells are exceedingly heterogeneous. CD8^+^ T cells are pivotal in eliminating tumor cells, and their dysfunction and reduced numbers contribute to GC immune tolerance. Toll-like receptor 2 is downregulated in CD8^+^ T cells of patients with GC. Additionally, the activation of toll-like receptor 2 can increase the expression of perforin and granzyme B inside CD8^+^ T cells and enhance CD8^+^ T cell cytotoxicity (28). The chromatin status of tumor-specific T cells is related to their dysfunction. Interestingly, patients with more open chromatin in their circulating CD8^+^ T cells tend to have longer survival times compared to those with closed chromatin (29, 30). T cells are tumor immune executors and perform the function of killing tumors in a straightforward manner. CD8^+^ T cells are dysfunctional and depleted in response to immune tolerance induced by the TME. Therefore, the prevailing solution for improving immune tolerance is to reverse immune tolerance, restore the quantity, reduce the extent of invasion, and recover CD8^+^ T cell function.

Regulatory T cells (Tregs) play a crucial role in forming an immunosuppressive environment and suppressing anti-tumor immune reactions (31). In an *in vitro* 3D organ model, early-stage intestinal-type GC is characterized by abundant Tregs, which have the capacity to boost the growth of tumor cell spheres by triggering the expression of interleukin-2rα and stimulating the mitogen-activated protein kinase signaling pathway (32). The degree of infiltration of tumor necrosis factor receptor 2 (TNFR2) positive Tregs increases with GC progression, serving as both a prognostic marker and an independent risk factor for GC. Activating the TNF-α/TNFR2 pathway facilitates Tregs function and stimulates their immunosuppressive phenotype (33). Accordingly, Tregs that infiltrate GC tissues play a pivotal role in disease progression by triggering immune tolerance. Targeting the suppression of Treg generation or function may alleviate immune tolerance, thereby delaying or treating the disease more effectively.

### 2.4 Natural Killer cells

NK cells can eliminate target cells directly and identify tumor cells that CD8^+^ T cells cannot identify. The level of NK cell infiltration in tumors and external blood is actively linked to the prognosis of patients with GC (34, 35). NK cell dysfunction is mainly caused by the upregulation of inhibitory receptors and downregulation of activating receptor signaling pathways in the TME. Death-associated protein kinase 1 (DAPK1), a tumor suppressor from the DAPK family, inhibits the IKKβ/CSN5/PD-L1 axis, enhancing the killing ability of NK cells and inhibiting immune evasion in GC (36). Cancer cell-derived prostaglandin E2 promotes NK cell apoptosis and inhibits proliferation (34). Furthermore, Pleckstrin-2 upregulates the expression of membrane-type 1 matrix metalloproteinase and induces major histocompatibility complex class I chain-related A shedding, ultimately reducing the sensitivity of GC cells to NK immune surveillance and promoting immune escape (37). Furthermore, a recent study found that L-kynurenine, derived from GC cells, induces ferroptosis in NK cells, resulting in their loss in the TME. This introduces a novel mechanism contributing to the decline in NK cells (38).

## 3 Heterogeneity of the tumor microenvironment

Researchers have used single-cell RNA sequencing (scRNA-seq) to construct a single-cell atlas that includes 32,332 cells from patients with gastric antrum mucosal biopsies covering a range of precancerous lesions and early GC (39). The analysis showed that glandular mucous cells tended to adopt an enteroid stem cell phenotype during metaplasia. In addition, OR51E1 serves as a marker of distinctive endocrine cells in early malignant lesions. Similarly, Hes family BHLH transcription factor 6 may identify pregoblet cellular clusters and contribute to the recognition of early metaplasia. Sathe et al. conducted scRNA-seq on seven patients with GC and one patient with intestinal metaplasia, validating their findings through multiple immunofluorescence assays (40). They found that the GC TME was prominently abundant in macrophages, stromal cells, dendritic cells (DCs), and Tregs.

Macrophages are transcriptionally heterogeneous and inconsistent with the M1/M2 paradigm. Tumor-DCs have a distinctive gene expression program compared with peripheral blood mononuclear cells. Costimulatory molecules or multiple immune checkpoints are produced by cytotoxic T cells, helper T cells, Tregs, and NK cells. Li et al. conducted scRNA-seq analysis on nine untreated patients with non-metastatic GC and found that Tregs were abundant in gastric tumor tissues, and the expression of the immunosuppression-related genes *DUSP4, LGALS1, TNFRSF4, LAYN*, and *IL2RA* was increased. The absence of individual exhausted CD8^+^ T cell clusters was also observed, as well as low expression of the exhausted markers HAVCR2, PDCD1, LAG-3, CTLA4, and TIGIT in these cells, which might function as molecular-level evidence for the restricted efficacy of immunotherapy in patients with GC (41). Likewise, CD8^+^ T cells have been shown to downregulate the interferon regulatory factor 8 transcription factor in the GC TME, and the expression level of interferon regulatory factor 8 in blood CD8^+^ T cells was associated with a more advanced stage (42).

Research on single-cell gene expression has revealed extensive reprogramming of multiple cell factors within the TME of GC. Cell remodeling encompasses variations in transcriptional status, cell number, and intercellular interactions. These advances enhance our understanding of tumor biology and open up possibilities for new target identification (43). In addition, the TME of patients with GC patients may undergo specific changes after treatment. Researchers have found that the levels of tumor-infiltrating lymphocytes change after neoadjuvant chemotherapy. In patients who responded to chemotherapy, the number of CD8^+^ T cells in the epithelial and stromal regions of the GC was significantly increased, the proportion of FOXP3-positive Tregs was significantly decreased, and the expression of PD-L1 on these Tregs was significantly reduced (44). Therefore, the TME in patients with GC is complex.

## 4 Immunotherapy in gastric cancer

A deeper understanding of the TME, especially TIME has greatly facilitated immunotherapy for GC (45). Immunotherapy can trigger an immune response in patients with GC that kills tumor cells. Moreover, immunotherapy has shown strong efficacy and tolerable toxicity compared with traditional therapies. Therefore, novel therapeutic strategies for GC are becoming increasingly popular. Antibody-drug conjugates (ADCs) combine the anti-tumour activity of antibodies with cytotoxicity of chemotherapeutic agents, and provide a novel and promising direction for patients with GC.

### 4.1 Immune checkpoint inhibitors

Programmed death 1 (PD1) is a co-inhibitory molecule found on the cell membranes of immune cells such as T cells, B cells, and myeloid cells. The interaction between programmed death ligand 1 (PD-L1) and PD1 initiates an immune response process (46, 47). PD-L1 is abundantly expressed in many malignant tumors and is also detected in GC, especially in patients with Epstein-Barr virus infection or microsatellite instability (48).

Immune checkpoint inhibitors (ICIs) have been developed and studied in preclinical and clinical settings. Nivolumab, a PD-1 inhibitor, is one kind of monoclonal antibody that was approved by the United States Food and Drug Administration (FDA) in 2014 for the management of advanced GC. Preliminary results from stage III clinical experiments in more than 40 nations in Asia have shown that nivolumab can significantly improve patient survival rate compared to a placebo. The overall 12-month survival rate for patients with stomach tumors treated with nivolumab was 26.2%, compared with 10.9% for patients treated with placebos (49). At present, nivolumab has been applied as a novel method for the therapy of recurrent and advanced GC (50, 51). In a multicenter, randomized, phase III trial, nivolumab plus chemotherapy showed prolonged overall survival (OS) and progression-free survival (PFS) compared with chemotherapy alone, which makes this combination a promising first-line therapy for advanced gastric adenocarcinoma (52). In addition, camrelizumab, another PD-1 inhibitor, combined with concurrent chemoradiotherapy, results in an encouraging pathological response in locally advanced gastric adenocarcinoma (53). The phase II clinical trial initiated by Liu Lian’s team in April 2019 creatively combined camrelizumab with anti-angiogenesis drugs (apatinib) and chemotherapy drugs (S-1 ± oxaliplatin) for neoadjuvant or conversion therapy in patients with cT4a/bN+ GC. This approach demonstrated improved efficacy and feasibility, especially in microsatellite instability-high and PD-L1 positive patients (54).

Pembrolizumab is a potential inhibitor of PD-L1 in GC. Its efficacy has been evaluated during a stage II trial, where it demonstrated moderate side effects as well as high anti-tumor activity (55). In a previous stage II clinical experiment, pembrolizumab monotherapy was administered to patients with gastroesophageal or advanced-stage GC who had undergone second-line therapy and showed promising activity with persistent responses (56). In a phase 3 interventional study, pembrolizumab provided a clinically meaningful effect in patients with PD-L1 combined positive score of 10 or greater or with microsatellite instability–high tumors (57). It is believed that PD-L1 inhibitors promote GC therapy by activating DCs, T lymphocytes, and NK cells, thereby resulting in GC destruction. Avelumab exhibited remarkable tolerability compared to chemotherapy in a stage III experiment in patients with advanced GC. Single-agent avelumab as a third-line therapy did not enhance OS or PFS, but it exhibited a more controllable security profile compared to chemotherapy (58). Similarly, in a recent randomized controlled clinical trial, atezolizumab showed no corresponding clinical activity in patients with GC, but showed a good safety profile. Atezolizumab combined with PEGylated recombinant human hyaluronidase (n=13) showed a confirmed objective response rate (ORR) of 0%, versus 16.7% in the control group (n=12). Notably, the incidence rates of grade 3 and 4 adverse events were 30.8% and 75.0%, respectively (59).

Cytotoxic T-lymphocyte-associated protein-4 (CTLA-4), a checkpoint molecule, is mainly expressed in Tregs and activated T cells, playing a pivotal role in maintaining immune homeostasis (60, 61). Clinical evaluations have been carried out for the CTLA-4 inhibitors ipilimumab and tremelimumab in patients with GC. A stage II study including 114 patients was performed to assess the safety and efficacy of ipilimumab monotherapy in patients with locally advanced/metastatic stage gastric or gastroesophageal cancer. The results of this study found that ipilimumab did not enhance immune-related PFS compared with the current best supportive treatment. Ipilimumab treatment resulted in treatment-associated adverse events in 72% (41/57) of patients, compared to 56% (25/45) in the group receiving active optimally supportive treatment (62). Similarly, tremelimumab, whether administered as monotherapy or in combination with durvalumab, showed a subdued therapeutic response, with no significant enhancement in ORR and 6-month PFS (63). Therefore, ICIs do not provide clinical benefits to all patients. In fact, extensive clinical experiments have shown that less than 30% of patients with GC respond positively to immunotherapy (64).

The advent of ICIs has marked a significant breakthrough in the treatment of advanced GC, demonstrating notable anti-tumor effects. However, the efficacy and toxicity of ICIs have limited their widespread clinical use (46, 65).

### 4.2 Adoptive cell therapy

Tumor cells can produce inhibitors that suppress the immune response, such as prostaglandin E2, TGF-β, and interleukin-10, as well as LAG-3, thereby leading to immune evasion. Adoptive cell transfer (ACT) therapy is an efficient strategy for treating GC in patients whose immune systems struggle to recognize or respond to tumor cells. ACT uses various immune cells, such as tumor-infiltrating lymphocytes (TILs), cytokine-induced killer (CIK) cells, and NK cells, to trigger efficient immune clearance of tumor cells (66, 67). In a randomized trial of patients with advanced GC, researchers found that TILs acted as an independent protective prognostic factor and that combination with chemotherapy could prolong survival time (68). Moreover, CIK cells produce cytokines and chemokines that regulate and enhance immune responses. Research on CIK cells has revealed their potent anti-tumor activity, especially when combined with classical adjuvant therapy (69). A prospective study of 63 patients with advanced GC showed that the combination of dendritic cell-CIK with S-1 plus cisplatin resulted in promising PFS and OS (70). Furthermore, the adverse effects and toxicity of CIK cell treatment are moderate and easy to control (69, 71). NK cells are activated as anti-tumor agents via the loss or downregulation of major histocompatibility complex I molecules, through which tumor cells can escape CTL recognition (72). Abdolahi et al. demonstrated that NK cells exhibit therapeutic cytotoxicity *in vitro*, and the combination of anti-PD-1 (nivolumab) improved the therapeutic efficacy of NK cells, which may contribute to the upregulation of CD69 and NKG2D (73). The efficacy and safety of ACT have been demonstrated in several clinical studies; however, specific regimens, doses, and antigens require further studies (74).

### 4.3 Chimeric antigen receptor T cell therapy

CAR T-cell therapies have been developed to treat disparate categories of carcinomas, such as acute lymphoblastic leukemia and various solid carcinomas (75). CAR consists of extracellular single chain variable fragments targeting multiple tumor-associated antigens (TAAs), an intracellular CD3 region, a transmembrane segment, and a costimulatory domain, such as the intracellular domains of CD28 or CD3ζ plus 4-1BB (76). When CAR specifically binds to TAAs on target tumor cells, CD3ζ and the costimulatory domain are stimulated and trigger a T cell phosphorylation cascade, resulting in transcription of genes encoding cytokines, release of cytotoxic particles, and cellular proliferation (77). Biomarkers, including mucin 1, claudin 18.2 (CLDN 18.2), human epidermal growth factor receptor 2 (HER2), NK receptor group 2, epithelial cell adhesion molecule, mesothelin (MSLN), and carcinoembryonic antigen, play pivotal roles in the diagnosis and progression of GC. Furthermore, CAR T cell therapy can efficiently target these biomarkers in GC therapy (78).

HER2 is an overexpressed surface antigen in GC cells, and HER2-positive GC is commonly multidrug-resistant, thus inhibiting the anti-tumor capacity of conventional drugs (79). Research on HER2-directed CAR T therapy has shown a high affinity for GC cells, and researchers have reported that amplified CAR T cell expression increases central memory phenotypes (80). Moreover, this expression was stimulated by the specific recognition of the HER2 antigen in an MHC-independent manner and efficiently eliminated patient-derived HER2^+^ GC cells. In the HER2^+^ xenograft GC model, CAR T cells showed significantly improved tumor suppressant ability, prolonged long-term prognosis, and enhanced targeting properties compared with non-transduced T cells. The pelletizing capability and tumorigenicity of GC stem cell-like cells, which release HER2 and CD44 proteins, were also suppressed. MSLN, a 40 kDa membrane protein, is expressed in normal mesothelial tissues and is highly expressed in GC. Jiang et al. described the effectiveness of anti-MSLN CAR T cells in GC through various *in vitro* and xenotransplantation mouse models. It was also demonstrated that local peritumoral administration improved CAR T-cell invasion and efficacy compared to systemic intravenous administration (81). CLDN 18.2 is the second isomer of claudin 18 on the external cell membrane and is expressed in differentiated gastric mucosal epithelial cells and in 70% of primary gastric adenocarcinomas and metastases (82). Engineered CLDN 18.2-specific CAR T cells exhibited robust persistence in the body and effectively infiltrate cancer tissue in mice without causing toxicity (83). They demonstrated substantial tumor regression, both partial and complete, in a xenograft model utilizing tumors derived from ClDN18.2-positive GC patients. These CAR T cells not only persisted well but also efficiently homed in on tumors, where they targeted and lysed CLDN18.2-expressing mouse cells, all without causing significant harm to normal organs, such as the gastric tissue in mice. Nevertheless, the clinical application of CAR T cell therapy faces numerous challenges (84). The utilization of autologous T cells is costly and requires a lengthy production course, in contrast to currently adopted approaches. There are many issues regarding the potency of CAR T cells in solid tumors, such as the heterogeneity of tumor antigens, tumor invasion, and the stability and proliferation of T cells within the tumor (78). One significant challenge is the immunosuppressive TME, which significantly impairs T cell function through the overexpression of inhibitory receptors, leading to rapid therapeutic cell depletion. Additionally, the diverse array of cells present in the TME, including TAMs, Tregs, myeloid-derived suppressor cells, and TANs, ultimately facilitates tumor immune escape (85).

### 4.4 Cancer vaccine

Cancer vaccines are a potential method of anti-tumor immunotherapy. Vaccines that target TAAs or tumor-specific antigens can accurately attack and destroy malignant cells with an elevated release of antigens, fulfilling sustained tumor elimination via immune memory (86, 87). Messenger RNA (mRNA) vaccines carry genetic data on antigens, swiftly translating them into proteins to trigger a robust and widespread immune response, ultimately leading to tumor cell reduction (88). Cafri et al. developed a process utilizing TILs to identify the expression of specific immunogenic mutations in tumors, conducted a clinical trial on mRNA vaccines, and found that the vaccine could induce mutation-specific T cell reactions against new epitopes that were not detected prior to vaccination (89). In addition, peptide vaccines comprising multiple cancer-testis antigens, such as forkhead box protein M1, upregulated lung cancer 10, KIF20, and DEPDC1, can safely activate specific T cell responses in patients with advanced GC (90). However, the prognostic benefits of this peptide vaccine were not significant.

Compared to other categories of immunotherapy, cancer vaccines theoretically offer a treatment that is specific, secure, and well tolerated. However, because of the variety of tumor antigens and relatively poor immune responses, the conversion of cancer vaccines into efficient treatments remains challenging (91). An increasing number of clinical studies have been conducted, particularly on personalized vaccines, and the development of vaccines for patients with GC is encouraging.

### 4.5 Antibody-drug conjugates

Recently, ADCs have emerged as potential anti-tumor agents, offering a new avenue for personalized cancer therapy (92). ADCs are composed of cytotoxic drugs cross-linked to monoclonal antibodies to target antigens that are expressed at a higher level in tumor cells than in normal cells. ADCs provide an optional targeting system for cytotoxic drugs and improve clinical therapeutic indicators (93). Because patients with HER2^+^ GC are still unable to benefit from trastuzumab, varieties of HER2-directed therapeutic innovative methods are being investigated, such as the potential antibody−drug combination trastuzumab-deruxtecan (T-DXd), for patients who have progressed from previous trastuzumab therapy. In a dose-expansion and dose-escalation stage I trial with an open-label design, 44 patients with gastroesophageal cancer or HER2-positive GC received no less than one dose of T-DXd-recommended dose-expansion therapy. This study demonstrated a manageable security profile for T-DXd with initial activity (94). Based on the results of the DESTINY-Gastric01 trial, T-DXd obtained FDA approval for the treatment of locally advanced, unresectable, or metastatic GC in adult patients previously treated with trastuzumab (95). Trastuzumab emtansine (T-DM1) is an ADC consisting of trastuzumab linked to the tubulin inhibitor emtansine (96). T-DM1 inhibits the HER2-derived signaling pathway, clarifies the extracellular domain of HER2 and activates antibody-dependent cellular cytotoxicity (97). An adaptive, open-label, randomized stageI/III phase study showed a median OS of 7.9 months for T-DM1 treatment. The moderate OS for taxane treatment was 8.6 months, with limited therapeutic effects despite a favorable safety profile (98). Nevertheless, Zhang et al. found that T-DM1 could induce apoptosis, yielding promising effects in HER2^+^ GC cells, and activate cytoprotective autophagy (99). Inhibiting autophagy might enhance the fusion of T-DM1 with lysosomes, expediting emtansine release. This study proposes a combination therapy strategy that could improve the therapeutic efficacy of T-DM1.

Disitamab vedotin (RC48), a novel anti-HER2 ADC, consists of hertuzumab and monomethyl auristatin E, coupled with a cleavable linker (100). In a phase I study of 57 patients with advanced HER2+ GC, RC48 demonstrated excellent clinical activity, promising anti-tumor activity, and an acceptable safety profile (101). RC48 also showed encouraging efficacy in patients treated with HER2-targeted drugs, achieving an ORR of 15.0%. In an open-label, multicenter, stage II study, RC48 verified clinically significant responses and survival benefits in intensively treated patients with locally advanced or metastatic HER2-overexpressing GC, achieving an ORR of 24.8% (102). In addition, RC48 exhibited anti-tumor effects in patients with low HER2 expression in GC (101).

Because ADCs are a combination of minor-molecule cytotoxic drugs and antibodies using synthetic linkers, resistance mechanisms for each component are constantly emerging. The mechanisms of antibody-related resistance (such as reducing cell-surface antigens) and payload-associated resistance (such as drug effector proteins) have been studied using other cytotoxic drugs and antibody-based treatments (103, 104). Wang et al. adopted HER2-positive N87 GC cells as materials for the sake of building T-DM1-resistant N87 cell lineages (N87-KR cells) (105). The kinetics of internalization, externalization, and binding were equal in N87 and N87-KR cells. N87-KR cells showed strong resistance to T-DM1, but were susceptible to mertansine and trastuzumab. Further studies have shown that abnormal vacuolar H^+^-ATPase activity reduced the metabolism of T-DM1, resulting in T-DM1 resistance in N87-KR cells. Different linkers in HER2-targeted ADCs, such as protease-cleavable linkers, may solve this resistance.

## 5 Discussion

GC is a malignant tumor characterized by rich and varied immune infiltrates, which provides various potential targets for immunotherapy. Immune cells, including TAMs, TANs, T cells, and NK cells, as well as their secreted chemokines and/or cytokines, play a pivotal role in GC immune programs and affect the efficacy of immunotherapy. Immunotherapy is rapidly developing and is a promising method for patients with advanced or metastatic GC, with ICIs featuring prominently various clinical trials. However, ICI monotherapy does not provide satisfactory curative effects in most patients with GC. In addition, immune cells or molecules in the TME may influence the efficacy of ICI combination therapy (106). To enhance the efficacy of new ICIs, it is crucial to delve into immune cells and factors in the tumor microenvironment using a single-cell approach. Recently, an increasing number of immune checkpoint modulators have been studied, including CD39, LAG-3, T cell immunoglobulin, and mucin domain-containing protein 3 (107). Furthermore, the deepening understanding of tumor heterogeneity presents another challenge to immunotherapy in patients with GC. The integration of personalized molecular data and molecular data from patients with GC could contribute to individualized therapy and ultimately prolong the survival of patients. Furthermore, other immunotherapies such as ACT, CAR T therapy, and cancer vaccines warrant further investigation. However, these exploratory studies often require mouse models of GC. While various mouse models exhibit critical human GC characteristics, they diverge significantly. Human GC tumorigenesis is a chronic process that involves years of inflammation and injury, resulting in early TP53 mutations. In contrast, mouse models primarily rely on the activation of oncogenic drivers, following a different molecular sequence of events (108, 109). Therefore, their reliability and clinical translatability remain unclear. Strengthening collaboration between clinical physicians and researchers is essential to leverage mouse models effectively. Efforts have been made to find suitable methods to predict the effect of immunotherapy. For example, multi-dimensional tumour-infiltrating immune cells signature, CT-based radiomics score relating with the neutrophil-to-lymphocyte ratio in the TIME, TMEscore R package and so on, these approaches may provide the impetus for precision immunotherapy, but further research is still needed (110–112).

## Author contributions

PM: Writing – original draft, Writing – review & editing. Q-hG: Writing – original draft. RS: Writing – original draft. T-fZ: Supervision, Writing – review & editing. YL: Supervision, Writing – review & editing. KD: Supervision, Writing – review & editing.

## Glossary

**Table:**

GC	gastric cancer
TME	tumor microenvironment
TIME	tumor immune microenvironment
NK	natural killer
TGF-β	transforming growth factor β
CAR	chimeric antigen receptor
TANs	tumor-associated neutrophils
Tregs	tumor regulatory cells
TNFR	tumor necrosis factor receptor
DAPK1	death-associated protein kinase 1
scRNA-seq	single-cell RNA sequencing
DCs	dendritic cells
ADCs	antibody-drug conjugates
PD-1	programmed death 1
PD-L1	programmed death ligand 1
ICIs	immune checkpoint inhibitors
FDA	the United States Food and Drug Administration
OS	overall survival
PFS	progression-free survival
ORR	objective response rate
CTLA-4	cytotoxic T-lymphocyte-associated protein-4
ACT	adoptive cell therapy
TILs	tumor-infiltrating lymphocytes
CIK	cytokine-induced killer cells
TAAs	tumor-associated antigens
CLDN 18.2	claudin 18.2
HER2	human epidermal growth factor receptor 2
MSLN	mesothelin
mRNA	message RNA
T-DXd	trastuzumab deruxtecan
T-DM1	trastuzumab emtansin
RC48	disitamab vedotin
N87-KR cells	T-DM1-resistant N87 cell lineages
